# Abbreviated MRI Protocols in Breast Cancer Diagnosis: A Narrative Review

**DOI:** 10.3390/jcm15176921

**Published:** 2026-09-07

**Authors:** Piotr Główczyk, Aleksandra Domżalska, Anna Hitnarowicz, Sylwia Grabowska, Katarzyna Steinhof-Radwańska, Mateusz Winder

**Affiliations:** 1 Students Scientific Association, Department of Oncological Radiology and Nuclear Medicine, Medical University of Silesia in Katowice, 40-514 Katowice, Poland; 2 Stobrawskie Medical Centre, 46-082 Kup, Poland; 3 Department of Diagnostic Imaging, Provincial Specialist Hospital No. 5 St. Barbara’s, 41-200 Sosnowiec, Poland; 4 Department of Oncological Radiology and Nuclear Medicine, Medical University of Silesia in Katowice, 40-514 Katowice, Poland

**Keywords:** abbreviated breast MRI, breast cancer, screening, diagnostic accuracy, dynamic contrast enhancement, diffusion-weighted imaging, dense breasts

## Abstract

**Background/Objectives**: Breast MRI is the most sensitive modality for breast cancer detection, but long examination times, high costs, and limited availability restrict its use. Abbreviated breast MRI (AB-MRI) aims to preserve clinically relevant information while reducing acquisition and interpretation times. This narrative review summarizes evidence through 2026 on AB-MRI protocols, diagnostic performance, clinical applications, limitations, and emerging developments. **Methods**: Clinical studies, reviews, guidelines, implementation studies, and technical investigations of AB-MRI for screening, surveillance, preoperative assessment, neoadjuvant chemotherapy response, postoperative imaging, ultrafast MRI, diffusion-weighted imaging, artificial intelligence, and patient experience were reviewed. **Results**: AB-MRI can achieve diagnostic performance comparable to full-protocol MRI in selected screening and surveillance settings while substantially reducing scan and reading times. Multireader data in women with extremely dense breasts showed no significant differences in sensitivity or specificity versus full MRI. However, performance depends on protocol composition and clinical setting; T2-weighted imaging and DWI may improve specificity or diagnostic performance in selected applications. Recent studies also support sequential screening, surveillance after breast cancer, and neoadjuvant response assessment, while limitations remain for invasive lobular carcinoma, small lesions, non-mass enhancement, and disease extent. Emerging strategies include ultrafast MRI, adaptive AI-based imaging, AI-generated gadolinium-free contrast enhancement, and DWI-based protocol optimization. **Conclusions**: AB-MRI is best viewed as a flexible, indication-specific strategy rather than a single universal protocol. It is particularly promising for supplemental screening and surveillance, but prospective multicenter validation and protocol standardization remain necessary.

## 1. Introduction

Breast cancer remains a major public health problem worldwide and is the most frequently diagnosed cancer among women [1,2]. Mammography, including digital mammography and digital breast tomosynthesis (DBT), remains the cornerstone of population-based screening. However, its sensitivity decreases in women with dense breast tissue, increasing the risk of occult malignancies [3,4]. Consequently, supplemental imaging strategies are increasingly considered according to individual breast density and cancer risk [5].

Breast magnetic resonance imaging (MRI) is the most sensitive breast imaging modality, particularly valuable in women at increased risk of breast cancer and those with dense breasts [5,6,7]. MRI also provides morphological and functional information useful for staging and treatment-response assessment. However, its broader implementation is limited by relatively long acquisition and interpretation times, cost, limited availability, and the need for intravenous contrast administration [6,7].

These limitations have stimulated the development of abbreviated breast MRI (AB-MRI) protocols, designed to preserve the high diagnostic performance of MRI while reducing examination and interpretation times [8,9]. The original approach focused on pre-contrast and early post-contrast T1-weighted acquisitions, subtraction images, and maximum-intensity-projection (MIP) reconstructions [9]. More recent concepts consider AB-MRI as a family of focused examinations, including FAST and ultrafast protocols, abridged protocols incorporating additional sequences such as T2-weighted imaging or diffusion-weighted imaging (DWI), and non-contrast approaches [10,11].

The increasing diversity of AB-MRI protocols indicates that their diagnostic performance depends not only on protocol composition but also on the clinical setting. The optimal balance between acquisition speed, sensitivity, specificity, and lesion characterization may differ between screening, surveillance, and diagnostic applications [10,11]. Recent systematic evidence confirms that AB-MRI can achieve diagnostic performance approaching that of full-protocol MRI in selected settings, although differences in sensitivity and specificity remain dependent on the protocol and indication [12,13].

The clinical role of AB-MRI is also expanding beyond initial supplemental screening. Current recommendations support MRI or AB-MRI as supplemental screening options in selected women according to breast density and individual risk [5]. Emerging evidence supports its use in sequential screening of women with dense breasts [14] and suggests potential applications in surveillance and treatment-response assessment [15,16].

Therefore, the aim of this study is to evaluate the clinical usefulness of abbreviated breast MRI protocols based on the available clinical evidence, with particular emphasis on diagnostic performance, protocol variability, acquisition and interpretation efficiency, and applicability across different clinical indications.

## 2. Materials and Methods

A comprehensive literature search was performed using the PubMed/MEDLINE, Scopus, and Web of Science databases. Additional relevant publications were identified through manual screening of reference lists. The primary AB-MRI literature search focused on studies published from January 2014 through August 2026. Earlier foundational publications relevant to breast MRI, breast density, and diffusion-based imaging were included for contextual background where appropriate. The final literature search was performed in August 2026. Only English-language publications were considered eligible for the primary evidence synthesis.

The search strategy combined Medical Subject Headings (MeSH) terms and free-text keywords including “abbreviated breast MRI”, “AB-MRI”, “fast breast MRI”, “FAST MRI”, “ultrafast MRI”, “breast magnetic resonance imaging”, “breast cancer screening”, “dense breasts”, “personal history of breast cancer”, “neoadjuvant chemotherapy”, “diffusion-weighted imaging”, “artificial intelligence”, “gadolinium-free MRI”, “patient experience”, and “protocol optimization”. Search terms were combined using the Boolean operators AND and OR, with combinations adapted to the syntax of each database.

Eligible publications included prospective and retrospective clinical studies, randomized or comparative studies, systematic reviews and meta-analyses, guideline or appropriateness documents, implementation studies, and relevant technical investigations evaluating abbreviated breast MRI. Studies addressing diagnostic accuracy, protocol composition, acquisition or interpretation time, lesion characterization, clinical implementation, artificial intelligence, ultrafast imaging, contrast-free approaches, or patient experience were considered eligible.

Publications unrelated to abbreviated breast MRI, conference abstracts without sufficient methodological or outcome information, editorials lacking relevant clinical or conceptual content, case reports, and studies focusing exclusively on conventional full-protocol MRI without relevance to abbreviated imaging were excluded from the evidence synthesis. Editorial or ongoing-study publications were retained only as contextual references and were not treated as outcome evidence.

Duplicate records were removed before study selection. Titles and abstracts were screened for relevance, followed by full-text assessment of potentially eligible publications according to the predefined inclusion and exclusion criteria. Any uncertainties regarding study eligibility were resolved by discussion among the authors.

Data extracted from the selected studies included publication year, study design, patient population, clinical indication, MRI protocol composition, acquisition time, interpretation time, diagnostic performance, comparison with full diagnostic protocol MRI, and principal conclusions. Particular attention was paid to pre-contrast and post-contrast T1-weighted imaging, subtraction images, MIP, T2-weighted imaging, DWI, DTI, ultrafast dynamic contrast-enhanced sequences, AI-assisted approaches, and patient-reported experience.

The included studies were grouped according to their primary clinical application: breast cancer screening, sequential screening and surveillance after breast cancer treatment, preoperative staging, assessment of response to neoadjuvant chemotherapy, postoperative evaluation, technical protocol optimization, artificial intelligence and contrast-free approaches, and implementation or patient-experience outcomes. Findings were synthesized descriptively because substantial heterogeneity in study design, patient populations, MRI acquisition protocols, and reported outcomes continued to preclude a single quantitative synthesis across all indications.

Because this study was designed as a narrative rather than a systematic review, a formal risk-of-bias assessment using tools such as QUADAS-2 was not performed. However, study design, population selection, reference standards, protocol heterogeneity, and potential spectrum or selection bias were considered qualitatively when interpreting the available evidence.

## 3. Development of Abbreviated MRI Protocols

### 3.1. From Full Diagnostic Protocol to AB-MRI

Breast MRI has become an essential imaging modality, complementing mammography and ultrasonography in clinical practice. Its primary applications include preoperative staging of confirmed breast cancer, screening in women at elevated risk, and assessment of response to neoadjuvant chemotherapy. Unlike mammography and ultrasound, MRI provides functional information, enabling evaluation of tissue vascularity and perfusion.

The foundations of contrast-enhanced breast MRI were established in the 1980s, when Heywang et al., as well as Kaiser and Zeitler, independently introduced this technique. Dynamic contrast-enhanced MRI relies on the administration of gadolinium-based contrast agents, which shorten T1 relaxation time and increase signal intensity on T1-weighted images. This approach is based on the biological process of tumor neoangiogenesis, which results in the formation of permeable microvasculature that facilitates rapid contrast agent leakage into the interstitial space, producing early and intense enhancement [10].

Although MRI technology has advanced considerably, this fundamental principle remains central to contemporary breast MRI. Modern protocols have evolved into multiparametric approaches, combining morphological and functional sequences to improve diagnostic performance [10]. FDP-MRI typically consists of a comprehensive multiparametric approach, including:

- T2-weighted imaging (with and/or without fat suppression);
- Pre-contrast T1-weighted imaging,
- Dynamic contrast-enhanced (DCE) sequences with 4–5 post-contrast phases;
- Frequent diffusion-weighted imaging (DWI) or additional delayed sequences [6,10].

Although it remains the reference standard for breast MRI, increasing efforts have focused on simplifying protocol design to improve examination efficiency while preserving diagnostic performance. The table below outlines a general comparison between standard and abbreviated approaches (Table 1).

### 3.2. Current Spectrum of Abbreviated Breast MRI Techniques

Abbreviated breast MRI (AB-MRI) comprises a heterogeneous group of shortened MRI examinations designed to reduce acquisition and interpretation time while preserving the diagnostic information required for a specific clinical task [9,10,11]. Protocols differ mainly in the number and timing of post-contrast acquisitions and in the inclusion of additional morphologic or functional sequences. Importantly, AB-MRI does not represent a single standardized protocol, and several related rapid MRI strategies should be distinguished [10,11].

FAST or single early post-contrast AB-MRI represents the simplest contrast-enhanced approach. The original FAST protocol consists of pre-contrast and first post-contrast T1-weighted acquisitions, subtraction images, and maximum-intensity-projection (MIP) reconstructions [9]. Its principal advantage is a marked reduction in acquisition and interpretation time. However, because evaluation is concentrated on the early post-contrast phase, information regarding delayed enhancement kinetics and detailed lesion characterization is limited [9,10].

Extended or abridged AB-MRI retains the basic early contrast-enhanced component while incorporating selected additional sequences, most commonly T2-weighted imaging, DWI, or additional post-contrast acquisitions [10,11,17]. These additions provide complementary morphologic, kinetic, or functional information and may improve lesion characterization and specificity, while maintaining a shorter examination than FDP-MRI. The exact composition of these protocols varies considerably between studies and clinical indications [10,13].

Within contrast-enhanced AB-MRI, multiphase abbreviated protocols incorporate more than one post-contrast acquisition while remaining shorter than conventional full dynamic examinations. By preserving additional kinetic information, these protocols may be useful when lesion characterization, disease extent, or treatment response is more important than rapid detection alone [10,18,19]. Their additional diagnostic value, however, appears to depend on the clinical setting, and current evidence does not support a predefined number of post-contrast phases as universally optimal [13,19].

Ultrafast DCE-MRI is a related but technically distinct contrast-enhanced technique. Rather than acquiring only a single early post-contrast image, ultrafast imaging repeatedly samples the initial passage of contrast with very high temporal resolution, allowing evaluation of early kinetic parameters such as time to enhancement [10,11,20]. This temporal resolution is generally obtained at the expense of spatial resolution compared with conventional high-resolution DCE acquisitions [10]. Ultrafast DCE-MRI may be incorporated into an abbreviated examination, but it should not be considered synonymous with conventional AB-MRI.

Unenhanced breast MRI represents a separate contrast-free strategy, relying primarily on DWI, often combined with T2-weighted or other non-contrast sequences [10,21,22,23]. Unlike contrast-enhanced AB-MRI, which derives diagnostic information largely from tumor vascularity and enhancement, unenhanced MRI is based predominantly on water diffusion and tissue characteristics. Its potential advantages include avoidance of gadolinium administration, but sensitivity may be reduced for small lesions, DCIS, and non-mass enhancement [21,22,23].

## 4. Clinical Evidence According to Indication

### 4.1. Supplemental Screening in Women with Dense Breasts

The evidence supporting AB-MRI is strongest in the supplemental screening setting, particularly in women with dense breasts, in whom the sensitivity of mammography is reduced by the masking effect of fibroglandular tissue. Current ACR recommendations recognize breast MRI and AB-MRI as potential supplemental screening options according to breast density and individual risk, including selected women with heterogeneously or extremely dense breasts [5]. The rationale for AB-MRI in this setting is therefore not to replace population-based mammography, but to provide a more accessible and time-efficient form of MRI-based supplemental screening.

The original FAST approach demonstrated that most of the diagnostic information required for cancer detection can be obtained from pre-contrast and early post-contrast images, allowing substantial shortening of MRI acquisition and interpretation [9]. More recent evidence confirms that abbreviated protocols may preserve much of the diagnostic performance of full-protocol MRI. In a multireader secondary analysis of the DENSE trial involving women with extremely dense breasts and negative mammography, sensitivity was 84.3% for the abbreviated protocol and 85.9% for full multiparametric MRI, while specificity was 73.9% and 75.8%, respectively, without significant differences. Importantly, interpretation time was reduced by approximately 50%, and scanning time by 70–80% [24]. These findings support the feasibility of substantial protocol reduction without a clear loss in diagnostic performance in this specific screening setting.

However, the available literature does not support assuming universal equivalence between AB-MRI and FDP-MRI. A 2024 systematic review and meta-analysis reported a higher pooled sensitivity for full-protocol MRI than for AB-MRI (95% vs. 86%), although no significant difference in specificity was observed [12]. More recently, a 2026 diagnostic test-accuracy meta-analysis including 40 studies and 14,266 patients demonstrated that performance varied according to both protocol composition and clinical setting. In screening populations, FAST maintained sensitivity comparable to FDP-MRI but showed lower specificity, whereas the addition of T2-weighted imaging increased specificity to a level not significantly different from FDP-MRI [13]. Thus, differences between published results appear to reflect, at least partly, heterogeneity in protocol design and study populations rather than a uniform difference between abbreviated and full MRI.

Importantly, the value of an imaging method for screening cannot be assessed on sensitivity alone. Specificity, recall or abnormal interpretation rates, positive predictive value, biopsy burden, cancer detection rate, and the consequences of false-positive findings are equally relevant. This is particularly important for very short protocols, in which reduced morphologic or kinetic information may increase the number of indeterminate findings. The recent meta-analytic evidence suggests that adding selected sequences, particularly T2-weighted imaging, may partly offset the specificity limitations of minimal FAST protocols [13]. Therefore, the shortest possible protocol is not necessarily the optimal screening protocol when downstream diagnostic burden is also considered.

Evidence from repeated AB-MRI examinations provides further clinically relevant information. Edmonds et al. evaluated 2585 supplemental AB-MRI examinations in women with dense breasts and found that the abnormal interpretation rate decreased substantially from 17.4% at baseline to 7.8% during subsequent screening rounds [14]. Cancer detection remained clinically meaningful, at 18.9 cancers per 1000 baseline examinations and 12.1 per 1000 subsequent examinations, while PPV values did not significantly deteriorate. Notably, all cancers detected during subsequent rounds were stage 0 or I and node-negative. These findings suggest that availability of prior AB-MRI examinations may reduce false-positive interpretations while maintaining detection of early-stage cancers, supporting evaluation of AB-MRI as a longitudinal rather than exclusively one-time supplemental screening strategy [14].

Nevertheless, screening results are not uniformly favorable across all populations. A prospective feasibility study in women aged 40–69 years with dense breasts found no additional cancers among those undergoing AB-MRI, while several BI-RADS 4 findings ultimately proved benign and resulted in additional ultrasound assessment and biopsies [25]. Although the cohort was relatively small and cannot establish lack of screening benefit, these findings illustrate the potential downstream consequences of false-positive AB-MRI findings and emphasize that diagnostic yield depends strongly on underlying cancer prevalence, population selection, and screening context.

Additional studies comparing AB-MRI with other imaging modalities in women with dense breasts generally support its high sensitivity, but their design should be considered when interpreting the results. For example, Ramli Hamid et al. reported similar sensitivity for AB-MRI and DBT (98.5% for both), with somewhat higher specificity and negative predictive value for AB-MRI [26]. However, most participants belonged to a diagnostic rather than a true population-screening cohort. Such enriched cohorts provide useful information on lesion detection but should not be directly used to estimate cancer detection, recall, or biopsy outcomes expected in asymptomatic screening populations.

Taken together, current evidence supports supplemental screening in women with dense breasts as the best-established clinical setting for AB-MRI, but it also demonstrates that screening performance extends beyond sensitivity. Minimal FAST protocols offer the greatest reductions in acquisition and interpretation time and appear capable of maintaining high cancer-detection sensitivity. However, reduced specificity and false-positive findings remain relevant concerns, and additional information from T2-weighted or selected dynamic sequences may improve characterization at the cost of modestly longer examinations [10,13]. At present, the evidence therefore supports an indication-adapted approach rather than a single universally optimal screening protocol. A short early contrast-enhanced protocol represents the best-studied foundation for dense-breast supplemental screening, whereas the decision to incorporate additional sequences should balance examination efficiency against specificity, lesion characterization, and downstream diagnostic burden.

### 4.2. Screening in High-Risk Women and BRCA Mutation Carriers

Women at high risk of breast cancer, including BRCA1/2 mutation carriers, represent a distinct screening population and should be considered separately from women undergoing supplemental screening primarily because of breast density. MRI is already an established component of intensified screening in this group, and current ACR criteria consider both full breast MRI and AB-MRI appropriate options for high-risk women regardless of breast density [5].

Available studies suggest that abbreviated protocols can substantially reduce acquisition and interpretation times while maintaining high cancer-detection performance in selected high-risk populations [8,27]. Panigrahi et al. reported that additional full-protocol sequences rarely changed BI-RADS assessment or biopsy management, suggesting that much of the information required for screening may be retained in shorter examinations [27].

Protocol composition, however, remains important. In BRCA1/2 mutation carriers, Naranjo et al. showed that adding T2-weighted imaging to a minimal abbreviated protocol increased specificity from approximately 72% to 83% while maintaining high sensitivity and negative predictive value [17]. These findings suggest that extreme protocol simplification may not be optimal in genetically high-risk women and that limited additional morphologic information may improve specificity.

More recent high-risk screening data have also shown high sensitivity and negative predictive value, with reported specificity of 85.7% and a cancer detection rate of 19.4 per 1000 examinations [28]. At the same time, the relatively low positive predictive value for biopsy highlights the importance of considering false-positive findings and downstream diagnostic procedures in addition to sensitivity.

### 4.3. Surveillance in Women with a Personal History of Breast Cancer

Women with a personal history of breast cancer (PHBC) represent a distinct surveillance population because of the ongoing risk of ipsilateral recurrence and second primary breast cancer. In this review, PHBC surveillance refers to periodic imaging of asymptomatic women after treatment and is distinguished from postoperative problem-solving MRI performed for a specific clinical or imaging abnormality.

Available evidence suggests that AB-MRI can provide clinically useful surveillance performance in selected women with PHBC while reducing examination time [16,29,30]. Reported studies have demonstrated high cancer-detection capability, including detection of small invasive cancers and DCIS, with many detected invasive cancers being early-stage and node-negative [29,30]. More recent data indicate that a basic FAST-type protocol may provide adequate performance in many surveillance examinations, with sensitivity around 81% and specificity approximately 88–91%, while the addition of ultrafast, delayed, T2-weighted, or diffusion-weighted sequences produced only modest improvements in diagnostic performance [16].

These findings suggest that extensive protocol expansion may not be necessary in every surveillance examination. However, performance may vary according to patient risk profile, breast density, prior tumor characteristics, and protocol composition, and current evidence remains insufficient to establish a single optimal AB-MRI surveillance protocol [16,31].

### 4.4. Preoperative Staging

The objective of preoperative breast MRI extends beyond detection of the index cancer and includes assessment of tumor extent, multifocal or multicentric disease, and additional ipsilateral or contralateral lesions that may influence surgical planning. These requirements place greater emphasis on spatial extent and lesion characterization and may limit the suitability of highly simplified abbreviated protocols [7,32].

Available studies report high sensitivity for AB-MRI in the preoperative setting, approaching 97–98% in selected cancer-enriched cohorts [32]. However, simplified protocols may be less reliable for small invasive cancers, DCIS, non-mass enhancement, invasive lobular carcinoma, and precise assessment of tumor extent [32,33,34]. High lesion-detection sensitivity should therefore not be interpreted as evidence that abbreviated protocols provide equivalent information for surgical planning.

Additional MRI-detected ipsilateral or contralateral lesions may alter the planned extent of breast-conserving surgery or raise consideration of mastectomy. At the same time, false-positive findings may lead to additional targeted imaging, biopsy, treatment delays, or potentially more extensive surgery [7]. Suspicious additional lesions that would alter definitive surgical management should therefore generally be confirmed before changing the operative approach.

Importantly, detection of additional lesions does not automatically translate into improved patient outcomes. Evidence specifically demonstrating that AB-MRI reduces positive margins, re-excision rates, local recurrence, or other surgical endpoints remains limited. Preoperative use of abbreviated protocols should therefore be interpreted primarily in terms of their ability to provide sufficient disease-mapping information rather than cancer detection alone.

### 4.5. Assessment of Response to Neoadjuvant Systemic Therapy

#### 4.5.1. General Performance of AB-MRI After NAC

Assessment of response to neoadjuvant systemic therapy represents a more demanding application of AB-MRI than cancer screening because the examination must identify not only the presence of residual enhancement but also the extent and pattern of residual disease. Treatment-related changes in tumor vascularity, cellularity, and morphology may alter enhancement characteristics and therefore reduce the reliability of protocols based exclusively on a single early post-contrast acquisition.

In the post-neoadjuvant setting, the optimal degree of dynamic contrast sampling remains uncertain. Earlier work suggested that preserving more than one post-contrast phase may improve assessment of residual disease, particularly when treatment alters enhancement kinetics and residual tumor enhances more slowly or heterogeneously. However, more recent studies indicate that highly abbreviated protocols based on an early post-contrast acquisition, particularly when combined with additional morphologic information such as T2-weighted imaging, may achieve diagnostic performance close to that of full-protocol MRI in selected patients [15,18,35,36]. These findings suggest that the benefit of extending the dynamic acquisition is not universal and may depend on the clinical endpoint, residual tumor pattern, and tumor biology rather than on a fixed number of post-contrast phases.

Recent multicenter evidence further suggests that protocol optimization may depend more on complementary functional information than simply on extending dynamic acquisition. Chen et al. evaluated abbreviated protocols combining T2-weighted imaging with different single post-contrast phases, with or without DWI. Addition of DWI significantly improved diagnostic performance, whereas extending the post-contrast acquisition did not provide additional benefit among DWI-based protocols [19]. Acquisition time remained 11–65% shorter than FDP-MRI, although DWI increased interpretation time [19].

#### 4.5.2. Influence of Tumor Subtype and Response Pattern

Breast cancer subtype is an important determinant of MRI performance after neoadjuvant systemic therapy because different molecular subtypes show distinct patterns of treatment response and residual enhancement. Consequently, HR-positive/HER2-negative, HER2-positive, and triple-negative breast cancers should not be considered a homogeneous imaging population when evaluating the performance of AB-MRI.

HR-positive/HER2-negative tumors generally show lower rates of pathologic complete response and may demonstrate more heterogeneous or incomplete regression. Residual disease can persist as scattered foci within the original tumor bed rather than as a single measurable mass, which may complicate assessment with highly abbreviated protocols. Conventional MRI evidence indicates lower sensitivity for pCR prediction in this subtype than in triple-negative tumors [37]. Therefore, preservation of sufficient morphologic information may be particularly important when residual disease rather than complete response is the primary clinical question.

HER2-positive tumors frequently demonstrate substantial response to systemic therapy, particularly when HER2-targeted treatment is used. Marked reduction or disappearance of enhancement may facilitate identification of treatment response; however, complete radiologic response does not necessarily exclude microscopic residual invasive disease or DCIS. Thus, even in tumors with pronounced treatment response, AB-MRI findings should be interpreted in the context of tumor biology and the limitations of imaging-based pCR prediction [35,37].

Triple-negative breast cancer (TNBC) typically exhibits higher pCR rates and often shows a more pronounced reduction in tumor enhancement after effective therapy. This may partly explain the relatively favorable MRI performance reported in this subgroup [37]. Preliminary subtype-specific evidence also suggests that rapid contrast-enhanced techniques may provide useful information in TNBC treated with NAC and immunotherapy, although evidence remains limited and ultrafast DCE-MRI should not be considered equivalent to conventional AB-MRI [38].

In addition to molecular subtype, the pattern of residual disease substantially influences MRI accuracy. Tumors may demonstrate concentric shrinkage, in which the residual lesion remains relatively well defined, or a scattered/fragmented response, in which small residual tumor foci remain distributed throughout the original tumor bed. Scattered patterns are more frequently associated with extensive pretreatment non-mass enhancement and are more difficult to quantify using a single lesion diameter [39]. Such patterns may be particularly challenging for simplified protocols with limited morphologic or kinetic information.

Residual DCIS and non-mass enhancement represent additional sources of diagnostic uncertainty. DCIS may show weak, heterogeneous, or absent enhancement after systemic therapy, and residual in situ disease may therefore be underestimated even when invasive tumor enhancement has disappeared. A systematic review found that only a proportion of residual DCIS remained detectable by enhancement on post-treatment MRI [40]. This is clinically relevant because diagnostic performance also depends on the definition of pCR—whether residual DCIS is permitted or classified as residual disease. Lo Gullo et al. addressed this issue by evaluating pCR using definitions both with and without residual DCIS [15].

Overall, post-NAC AB-MRI performance depends on both tumor subtype and residual disease pattern, and the current evidence does not support a single universal protocol.

### 4.6. Diagnostic and Post-Treatment Applications

Diagnostic imaging of the treated breast represents a distinct clinical problem from routine surveillance in women with a personal history of breast cancer (PHBC). After breast-conserving surgery and other local treatments, structural distortion, fibrosis, edema, seroma, fat necrosis, and inflammatory changes may produce morphologic or enhancement features that overlap with recurrent malignancy. In this setting, lesion characterization and specificity become particularly important, and the diagnostic objective extends beyond simply detecting early enhancement.

This distinction is relevant when considering protocol abbreviation. Minimal FAST-type protocols are primarily optimized for rapid detection of enhancing abnormalities, whereas evaluation of the postoperative breast may require additional information to distinguish recurrent disease from benign treatment-related changes. T2-weighted and diffusion-based techniques can provide complementary tissue characterization, potentially improving diagnostic confidence when enhancement alone is nonspecific [10,11,41]. Evidence from postoperative applications suggests that diffusion-derived information may improve differentiation of recurrent malignancy from benign post-treatment findings, although the available studies remain limited [41]. Reduced specificity is a particular concern in the postoperative breast because fibrosis, edema, and inflammatory changes may overlap with malignancy on early contrast-enhanced imaging.

By contrast, the evidence base is broader for routine surveillance of women with PHBC. Several studies have shown that abbreviated protocols can provide clinically useful cancer detection while reducing examination burden [16,29,30]. More recent data suggest that a basic FAST approach may already provide reasonable surveillance performance and that the incremental benefit of systematically adding ultrafast, delayed, T2-weighted, or diffusion-weighted sequences may be relatively modest [16]. However, these results should not be extrapolated directly to problem-solving examinations in symptomatic patients or in women with a specific suspicious postoperative finding. The ongoing multicenter KBCSG-27 trial further illustrates that AB-MRI versus full-protocol MRI in PHBC surveillance remains an active area of prospective investigation rather than a fully resolved clinical question [42].

Short abbreviated protocols appear promising for routine surveillance, whereas diagnostic evaluation of suspected recurrence may require greater morphologic and functional information. In particular, the presence of postoperative distortion or equivocal enhancement may favor incorporation of T2-weighted or diffusion-based sequences rather than reliance on a minimal early post-contrast protocol alone [7,10,11,41]. Current evidence is insufficient to define a standardized abbreviated protocol for postoperative problem-solving or to establish AB-MRI as a general replacement for FDP-MRI in this setting.

Diagnostic performance estimates should not be compared directly across clinical indications because of differences in disease prevalence, lesion spectrum, study design, protocol composition, and reference standards. Estimates from cancer-enriched or selected diagnostic cohorts should not be interpreted as equivalent to those obtained in population-based screening or surveillance cohorts. All studies summarized in Table 2 used contrast-enhanced MRI protocols.

As summarized in Table 2, AB-MRI protocols and diagnostic performance vary substantially across clinical indications. Evidence is most consistent for supplemental screening, whereas high-risk screening and PHBC surveillance are supported by a growing but less extensive evidence base. Evidence for preoperative staging, neoadjuvant response assessment, and postoperative diagnostic applications remains more limited and heterogeneous. Where reported, AB-MRI acquisition times ranged from approximately 3 to 11.5 min, with substantial variation in protocol composition according to the clinical application.

## 5. Role of MRI Sequences in Abbreviated Protocols

Abbreviated breast MRI (AB-MRI) does not represent a single standardized protocol. Sequence selection varies according to the clinical indication, diagnostic objective, and acquisition strategy. Accordingly, the role of individual sequences should be considered in terms of their contribution to lesion detection, characterization, and overall diagnostic performance. Differences in study populations, protocol composition, and reference standards limit direct comparison across studies.

For clarity, contrast-enhanced AB-MRI should be distinguished from ultrafast DCE-MRI and from unenhanced DWI-based MRI, which represent related but distinct imaging strategies.

### 5.1. Dynamic Contrast-Enhanced (DCE) T1-Weighted Imaging

DCE T1-weighted imaging is the core component of most contrast-enhanced AB-MRI protocols [9,47]. Commonly reported approaches include a pre-contrast acquisition followed by an early post-contrast T1-weighted sequence, often with subtraction or maximum-intensity-projection reconstructions [9,10,11]. A single early post-contrast acquisition has demonstrated very high sensitivity, approximately 96% in selected study populations, suggesting that full dynamic analysis may not always be necessary when the primary objective is rapid lesion detection [9,12]. Subtraction images may further improve lesion conspicuity without substantially reducing sensitivity [9].

Selected studies have reported sensitivity and specificity approaching those of FDP-MRI using minimal single-phase protocols [9,12,47]. However, these findings should not be interpreted as universal equivalence, because diagnostic performance varies with clinical setting, disease prevalence, lesion spectrum, and study design. Very high sensitivity values derived from retrospective or cancer-enriched cohorts are particularly susceptible to spectrum and selection bias.

### 5.2. T2-Weighted Imaging

T2-weighted imaging is not consistently required for basic lesion detection but provides complementary morphologic information for lesion characterization [13,17]. It can help identify fluid, edema, cystic changes, seromas, inflammatory changes, and other benign or treatment-related findings. Its main contribution appears to be improved characterization and specificity rather than increased sensitivity. In selected studies, incorporation of T2-weighted imaging improved diagnostic confidence and specificity while maintaining high sensitivity [13,17].

T2-weighted imaging should therefore not be regarded as an obligatory component of every AB-MRI protocol. Its inclusion may be particularly useful when the clinical task extends beyond rapid lesion detection to differentiation of benign and malignant findings.

### 5.3. Diffusion-Weighted Imaging (DWI)

DWI provides functional information based on water diffusion and tissue cellularity and may complement contrast-enhanced MRI [21,22,23]. Malignant lesions commonly demonstrate restricted diffusion and low apparent diffusion coefficient values. It performs better for larger lesions, whereas sensitivity may decrease for tumors smaller than 10 mm [21]. Lower sensitivity has also been reported for DCIS, mucinous tumors, necrotic triple-negative cancers, invasive lobular carcinoma, and non-mass enhancement [21,22,23].

DWI may be used either as an additional sequence within contrast-enhanced AB-MRI or as part of a distinct unenhanced MRI strategy. DWI-based imaging should therefore not be considered equivalent to conventional contrast-enhanced AB-MRI and does not currently replace contrast-enhanced breast MRI.

### 5.4. Ultrafast MRI and Kinetic Analysis

Ultrafast DCE-MRI is a technically distinct rapid contrast-enhanced technique characterized by very high temporal resolution during the earliest phase of contrast enhancement [10,11,20]. It may be incorporated into an abbreviated examination but should not be considered synonymous with conventional AB-MRI.

Ultrafast imaging enables assessment of kinetic parameters such as maximum slope, time to enhancement, and arteriovenous index. Selected studies have demonstrated high sensitivity and specificity for malignant lesion detection [20], although performance depends on acquisition technique, temporal and spatial resolution, contrast administration, and patient population.

The main limitations include incomplete morphologic assessment, overlap between benign and malignant enhancement patterns, and technical dependence on acquisition parameters. Ultrafast DCE-MRI should therefore be regarded as a complementary rapid technique rather than a replacement for conventional DCE-MRI or FDP-MRI.

## 6. Emerging Developments

### 6.1. Toward Adaptive and Indication-Specific MRI Protocols

Recent evidence supports viewing AB-MRI as a modular rather than fixed examination, in which sequence selection may be adapted to the clinical indication and the diagnostic information required [10,13]. Minimal early contrast-enhanced protocols may be sufficient when rapid cancer detection is the primary objective, whereas additional morphologic, dynamic, or diffusion-based information may be valuable when lesion characterization, disease extent, or treatment response is clinically important [14,16,19]. Future protocol development may therefore increasingly focus on adaptive rather than uniformly abbreviated examinations.

### 6.2. Artificial Intelligence and Contrast-Free MRI

Artificial intelligence is being investigated as a potential tool for further optimization of breast MRI acquisition and interpretation. AI has been proposed as a decision-support tool for adaptive scanning, in which additional sequences are acquired only when an initial abbreviated examination is flagged as suspicious [48].

Another emerging direction is contrast-free breast MRI. These approaches primarily rely on DWI with or without T2-weighted and other unenhanced sequences and should be considered separately from conventional contrast-enhanced AB-MRI. Their potential advantages include avoidance of gadolinium administration and shorter examinations, but current evidence does not support replacement of contrast-enhanced MRI across clinical indications [21,22,23]. More experimental approaches combine artificial intelligence with unenhanced imaging. Wang et al. generated synthetic contrast-enhanced images from precontrast T1-weighted and DWI data and reported improved cancer detection when these images were added to unenhanced MRI [49]. However, these findings derive from a retrospective technical study and support feasibility rather than clinical replacement of established contrast-enhanced MRI.

### 6.3. Implementation Considerations

The clinical value of AB-MRI depends not only on diagnostic performance but also on practical implementation. Shorter examinations may increase scanner throughput and potentially create additional imaging capacity, but the magnitude of these benefits depends on local infrastructure, examination volume, workflow, reader expertise, and patient selection. Effects on overall healthcare costs and access to MRI have not yet been sufficiently established.

Reader training is particularly relevant because highly abbreviated examinations provide less morphologic and kinetic information than FDP-MRI. Structured training and feedback may improve interpretation performance [50]. Patient acceptance is also important; although shorter examinations may improve tolerability, contrast administration, anxiety, and examination-related discomfort remain potential barriers [51].

Therefore, scanner throughput, reader training, patient acceptance, contrast-agent strategy, and emerging AI-assisted approaches should be considered together when evaluating future implementation of AB-MRI [48,50,51,52]. These developments remain strategies for optimization rather than established replacements for conventional breast MRI workflows.

### 6.4. Indication-Specific Framework for AB-MRI

The protocol compositions shown represent an evidence-informed synthesis of commonly evaluated approaches rather than standardized clinical recommendations (Table 3). Sequence selection should remain tailored to the clinical indication, diagnostic objective, patient characteristics, local expertise, and available resources. Evidence-maturity categories are qualitative and do not represent a formal GRADE assessment.

## 7. Limitations of AB-MRI and of the Current Evidence

### 7.1. Technical and Diagnostic Limitations

The principal limitation of AB-MRI arises from the same feature that provides its practical advantage: the reduction in the number of acquired sequences. Although early contrast enhancement contains sufficient information for detection of many breast cancers, protocol simplification inevitably reduces the amount of morphologic, kinetic, and functional information available for lesion characterization [9,10,11].

This trade-off may particularly affect specificity. Minimal FAST protocols can detect enhancing abnormalities rapidly, but limited characterization may increase the number of indeterminate or false-positive findings. Recent systematic evidence supports this distinction: in screening settings, FAST maintained high sensitivity but showed lower specificity than FDP-MRI, whereas incorporation of T2-weighted imaging partly mitigated this limitation [13]. Thus, the shortest protocol should not automatically be regarded as the diagnostically optimal protocol, particularly when false-positive findings may lead to additional imaging or biopsy.

Diagnostic limitations are also lesion-dependent. Small invasive cancers, low-grade DCIS, non-mass enhancement (NME), and lesions with delayed or weak enhancement may be less conspicuous when imaging is restricted to the earliest post-contrast phase [12,32,33]. The 2024 meta-analysis identified small lesions and NME as important challenges for abbreviated protocols, while pooled sensitivity was lower for AB-MRI than for FDP-MRI, emphasizing that high sensitivity reported in individual studies should not be generalized to every lesion type or population [12].

Invasive lobular carcinoma (ILC) deserves particular consideration because its infiltrative growth pattern and potentially delayed enhancement may limit detection or assessment with highly abbreviated examinations [33]. Although individual studies have reported high sensitivity for AB-MRI in ILC, specificity may be lower than with the full protocol, and the available evidence remains limited [34]. Similar concerns apply to DCIS, in which enhancement may be subtle, delayed, or predominantly non-mass-like.

Finally, cancer detection and accurate assessment of disease extent are not equivalent diagnostic objectives. A minimal protocol may identify the dominant lesion while providing less information on multifocality, multicentricity, lesion distribution, or scattered residual disease. This limitation is particularly relevant in preoperative staging and treatment-response assessment, where underestimation of extent can directly affect clinical management [32]. The original evidence base already indicates that very restricted approaches such as MIP alone can underestimate disease extent despite relatively high lesion-detection sensitivity.

### 7.2. Protocol and Population Heterogeneity, and Spectrum Bias

A major challenge in interpreting the AB-MRI literature is the substantial heterogeneity in both imaging protocols and study populations. The term AB-MRI encompasses examinations ranging from minimal FAST protocols using a single early post-contrast acquisition to extended protocols incorporating T2-weighted imaging, DWI, or additional dynamic phases. Ultrafast DCE-MRI is also included in some studies despite representing a technically distinct high-temporal-resolution acquisition strategy. Acquisition times therefore range from only a few minutes to approximately 10 min, and studies differ substantially in spatial resolution, timing of post-contrast acquisitions, use of subtraction or MIP images, and inclusion of additional functional sequences [9,10,11].

This technical variability has direct consequences for reported diagnostic performance. The recent diagnostic test-accuracy meta-analysis demonstrated that the relative sensitivity and specificity of AB-MRI and FDP-MRI changed according to the exact abbreviated protocol and clinical setting, and many protocol-specific subgroup analyses contained only a small number of studies [13]. Consequently, diagnostic estimates obtained for one FAST, extended, or multiphase protocol should not be extrapolated to AB-MRI as a single homogeneous technique.

An equally important source of heterogeneity is the clinical spectrum of the populations studied. Published cohorts include asymptomatic women undergoing population or supplemental screening, women at hereditary high risk, women with a personal history of breast cancer, patients with suspicious imaging findings, cohorts with biopsy-proven malignancy, preoperative populations, and patients undergoing neoadjuvant systemic therapy. The prevalence, size, histologic distribution, and conspicuity of cancer differ markedly among these groups.

Studies based on biopsy-proven or cancer-enriched cohorts are prone to spectrum and selection bias and should not be directly compared with population-based screening cohorts. In enriched diagnostic populations, cancers are typically more prevalent and may be larger or more conspicuous than cancers encountered in prospective screening. This can inflate apparent sensitivity and accuracy and can also make estimates of predictive values clinically non-transferable to lower-prevalence screening populations. Conversely, true screening cohorts include a much larger proportion of normal and benign examinations and therefore provide more informative estimates of specificity, recall, biopsy burden, and cancer detection rate.

This distinction is particularly important when interpreting reports of sensitivity approaching or reaching 100%. Such values may accurately describe the selected study sample but should not be interpreted as evidence of universal diagnostic equivalence between AB-MRI and FDP-MRI. The contrast between highly favorable results from selected cohorts and more conservative pooled estimates from meta-analyses illustrates the importance of population spectrum and study design [12,13].

Therefore, cross-study comparisons should account for clinical indication, population spectrum, protocol composition, and reference standard.

### 7.3. Limitations of the Available Evidence

The current evidence base has several methodological limitations that restrict the strength of clinical conclusions. A substantial proportion of published AB-MRI studies are retrospective, single-center investigations based on relatively small or selected populations. Although several larger and prospective studies are now available, robust prospective evidence remains concentrated primarily in screening, whereas data for preoperative staging, postoperative problem solving, and treatment-response assessment are considerably less mature.

Reference standards are also inconsistent. Some studies use histopathology for all included lesions, whereas others combine biopsy results with imaging follow-up or clinical surveillance. Follow-up duration and definitions of true-negative examinations vary, further complicating comparisons between studies. In treatment-response studies, even the definition of pathologic complete response may differ depending on whether residual DCIS is included, which can alter calculated diagnostic performance.

Outcome reporting is similarly heterogeneous. Sensitivity is reported more consistently than specificity, while recall rate, biopsy rate, PPV, cancer detection rate, interval cancer rate, and downstream diagnostic burden are frequently absent. This is particularly problematic in screening studies, where high sensitivity alone is insufficient to establish clinical utility.

Formal protocol standardization is currently lacking. Differences in scanner platforms, field strength, temporal and spatial resolution, contrast administration, sequence timing, interpretation criteria, and reader experience all introduce additional variability. Even recent meta-analytic subgroup estimates are limited by small numbers of studies for several specific protocol configurations, requiring cautious interpretation before clinical adoption [13].

Finally, the available literature provides limited evidence regarding whether reductions in acquisition and interpretation time translate into improved patient outcomes, cost-effectiveness, reduced biopsy burden, or broader access to MRI. These potential advantages are plausible but should not be assumed solely from shorter examination duration.

## 8. Discussion

The present review indicates that AB-MRI has progressed from an initial proof-of-concept approach to an increasingly investigated and selectively implemented strategy for shortening breast MRI examinations. Since the introduction of the first abbreviated protocol by Kuhl et al., accumulating evidence suggests that abbreviated protocols can preserve high sensitivity in selected clinical settings, particularly screening and surveillance. However, recent systematic reviews do not support universal diagnostic equivalence between AB-MRI and FDP-MRI [12,13]. These findings support the concept that early contrast enhancement contains substantial information relevant to breast cancer detection, while also indicating that the degree of acceptable protocol abbreviation depends on the clinical indication, protocol composition, and lesion characteristics [9,10,11,12,13].

One of the principal findings of this review is that the diagnostic performance of AB-MRI depends on the interaction between protocol composition, clinical indication, patient population, and lesion phenotype rather than on examination duration alone. Although the shortest protocols require only one pre-contrast and one early post-contrast acquisition, the addition of selected sequences, including T2-weighted imaging, DWI, or additional post-contrast information, may improve lesion characterization and specificity while preserving much of the time-saving advantage of abbreviated imaging [10,13]. Importantly, the incremental value of these sequences is not uniform across indications. Consequently, the concept of a single universal AB-MRI protocol appears unrealistic. Instead, the available evidence favors an indication-specific and modular approach in which protocol composition reflects the diagnostic task and the level of morphologic, kinetic, or functional information required.

The reviewed evidence indicates that the strongest evidence for AB-MRI currently relates to supplemental screening, particularly in women with dense breast tissue. Mammography has well-recognized limitations in dense breasts because overlapping fibroglandular tissue may obscure malignancies, reducing sensitivity. Studies in this setting demonstrate that substantial reductions in acquisition and interpretation time can be achieved while retaining high cancer-detection sensitivity [14,24]. Nevertheless, screening performance should not be judged by sensitivity alone. Specificity, abnormal interpretation or recall rates, biopsy rates, positive predictive value, cancer detection rate, interval cancers, and downstream diagnostic burden are also clinically relevant outcomes. Repeat-screening data suggest that abnormal interpretation rates may decrease when prior AB-MRI examinations are available, while cancer detection remains clinically meaningful [14].

High-risk women, including BRCA1/2 mutation carriers, represent a separate screening population and should not be conflated with average- or intermediate-risk women undergoing supplemental imaging because of breast density. Available studies support the feasibility of abbreviated protocols in high-risk screening, but protocol composition may influence specificity, with T2-weighted imaging providing additional value in some populations [17,27,28]. Current ACR criteria recognize MRI or AB-MRI as appropriate options in selected high-risk and density-based scenarios, but this should not be interpreted as evidence that AB-MRI can universally replace established full-protocol MRI surveillance [5].

AB-MRI should also be interpreted within the broader landscape of supplemental breast imaging rather than in isolation. DBT, ultrasound, contrast-enhanced mammography, and MRI address partly overlapping clinical needs, and their relative value varies according to breast density, individual risk, availability, contraindications, and local resources [5,7]. Therefore, shorter MRI acquisition alone does not establish superiority over competing modalities, and direct comparative and health-economic evidence remains important.

Beyond screening, this review highlights that the strength of evidence and the diagnostic requirements differ substantially among clinical indications. In women with a personal history of breast cancer, available data support AB-MRI as a promising surveillance strategy, but the evidence remains less mature than for dense-breast supplemental screening and prospective validation is ongoing [16,29,30,42]. Routine PHBC surveillance should also be distinguished from problem-solving evaluation of a suspicious postoperative finding, for which evidence supporting abbreviated protocols is considerably more limited.

Evidence for preoperative staging, postoperative problem solving, and assessment of response to neoadjuvant systemic therapy is more heterogeneous and does not currently support a single abbreviated protocol. In the preoperative setting, detection of additional lesions should not be equated with clinical benefit, because false-positive findings may result in additional biopsies or changes in surgical management without proven improvement in patient outcomes. After neoadjuvant therapy, diagnostic accuracy depends not only on protocol design but also on molecular subtype, residual DCIS, non-mass enhancement, and concentric versus scattered patterns of response [15,19,35,36,37,38,39,40]. Additional dynamic or diffusion-based information may be useful in selected patients, but current evidence does not support a universal requirement for a predefined number of post-contrast phases. These findings indicate that protocol simplification should not compromise the diagnostic objectives of individual clinical applications.

Another important observation is the increasing role of complementary morphologic and functional imaging in rapid breast MRI strategies. Although dynamic contrast-enhanced T1-weighted imaging remains the cornerstone of contrast-enhanced AB-MRI, T2-weighted and diffusion-based sequences may improve characterization or specificity when additional information is clinically required. Importantly, DWI-based unenhanced MRI and ultrafast DCE-MRI should not be treated as synonyms for AB-MRI. Unenhanced DWI-based MRI represents a distinct contrast-free strategy, whereas ultrafast DCE-MRI is a high-temporal-resolution contrast-enhanced technique that may be incorporated into an abbreviated examination [10,11]. Their potential advantages should therefore be interpreted according to the specific technique and indication rather than generalized to AB-MRI as a whole.

Despite these advantages, interpretation of the published literature is limited by substantial technical and methodological heterogeneity. Protocols differ in the number and timing of post-contrast acquisitions, spatial and temporal resolution, inclusion of T2-weighted or diffusion-based sequences, and interpretation criteria [12,13]. Equally important, the studied populations range from true screening cohorts to diagnostic, biopsy-proven, preoperative, and cancer-enriched populations. Studies based on biopsy-proven or cancer-enriched cohorts are susceptible to spectrum and selection bias and should not be directly compared with population-based screening cohorts. Very high or even 100% sensitivity reported in selected cohorts may therefore reflect, at least partly, disease prevalence and lesion spectrum rather than universally reproducible performance of AB-MRI.

This heterogeneity also helps explain why findings from individual studies and pooled analyses are not always concordant. While several selected cohorts report performance close to FDP-MRI, meta-analytic evidence shows that full-protocol MRI may retain a sensitivity advantage in some populations and that FAST-only protocols may have lower specificity [12,13]. Consequently, numerical estimates of sensitivity or specificity should always be interpreted together with the study design, clinical indication, cancer prevalence, lesion spectrum, and reference standard.

The findings of this review have several practical implications. Rather than replacing conventional breast MRI in every clinical setting, AB-MRI should be regarded as a flexible diagnostic strategy whose composition is adapted to the clinical question. Minimal protocols may be appropriate for screening and surveillance, whereas more comprehensive abbreviated protocols incorporating additional functional sequences are likely to provide greater diagnostic confidence in preoperative assessment, postoperative imaging, and evaluation of treatment response. Minimal early contrast-enhanced protocols appear best supported for supplemental screening, while selected additional sequences may be considered when characterization becomes more important. Evidence for surveillance is promising, whereas preoperative staging, postoperative problem solving, and treatment-response assessment remain less established indications. Such an individualized approach may optimize the balance between examination efficiency and diagnostic performance, but it should currently be regarded as an evidence-informed framework rather than a standardized clinical recommendation.

Implementation should likewise be considered cautiously. Shorter acquisition and interpretation times may increase scanner throughput, but they do not automatically translate into lower costs, wider access, fewer biopsies, or improved patient outcomes. Reader training, patient acceptance, contrast administration, local workflow, and scanner availability also influence real-world effectiveness [48,50,51,52]. Similarly, AI-assisted interpretation, adaptive imaging, synthetic contrast generation, and contrast-free approaches remain promising areas of development but should be distinguished from established clinical evidence.

This review has several limitations. As a narrative review, it is subject to potential selection bias and does not provide a quantitative synthesis of diagnostic performance. In addition, the included studies differed considerably regarding study design, patient populations, MRI systems, protocol composition, reference standards, and reported outcome measures, limiting direct comparison of results. Specificity and clinically relevant downstream outcomes were also reported inconsistently, and many studies were retrospective, single-center, or based on enriched populations. These limitations restrict the certainty and generalizability of conclusions regarding equivalence with FDP-MRI. Although the inclusion of recent studies and meta-analyses strengthens the currency of this review, the available evidence remains uneven across indications and does not permit definition of a single optimal AB-MRI protocol.

## 9. Future Research

Future research should prioritize prospective multicenter studies using standardized acquisition parameters, clearly defined clinical populations, and uniform reporting criteria [10,12,13,42]. Direct comparisons of different abbreviated protocols within specific clinical indications are needed to determine the optimal balance between examination time, sensitivity, specificity, and the level of diagnostic information required [10,13,16,19]. Further investigation into contrast-free MRI, artificial intelligence-assisted acquisition and interpretation, and ultrafast DCE-MRI is also warranted [20,21,22,23,48,49]. In particular, AI-generated synthetic contrast and adaptive AI-guided MRI represent promising approaches, but current evidence remains preliminary and requires prospective clinical validation [48,49]. Health-economic analyses should additionally determine whether reductions in acquisition and interpretation time translate into meaningful improvements in scanner throughput, cost-effectiveness, and access to breast MRI [7,10].

Ultimately, greater standardization of protocol composition, acquisition parameters, interpretation criteria, and outcome reporting will be necessary to facilitate comparison across studies and clinical implementation [10,11,12,13]. Development of consensus-based recommendations may therefore become appropriate as prospective evidence accumulates across individual clinical indications.

## 10. Conclusions

Taken together, current evidence supports AB-MRI most strongly as a time-efficient approach to supplemental breast cancer screening, particularly in women with dense breasts, where appropriately selected abbreviated contrast-enhanced protocols can preserve high cancer-detection performance while substantially reducing acquisition and interpretation time. Evidence in selected high-risk populations and PHBC surveillance is promising but remains less mature, whereas preoperative staging, postoperative problem-solving, and neoadjuvant treatment-response assessment are supported by more heterogeneous and indication-specific data and should not currently be regarded as established replacements for FDP-MRI. Overall, AB-MRI should be considered a modular, indication-specific strategy whose protocol composition is tailored to the diagnostic task; prospective multicenter validation, standardized outcome reporting, and health-economic evidence are needed before broader clinical implementation.

## Figures and Tables

**Table 1:** Characteristics of FDP-MRI and AB-MRI based on chosen features.

Feature	FDP-MRI	AB-MRI
Acquisition Time	Longer, typically ~15–30+ min	Shorter, commonly ~3–12 min; protocol-dependent
Post-contrast Phases	Multiple dynamic phases	One or a few selected phases; protocol-dependent
Primary goal	Comprehensive detection, characterization, and disease-extent assessment	Efficient lesion detection with task-specific characterization
Resource implications	Greater scanner and interpretation burden	Potentially improved throughput and reduced resource use

**Table 2:** Diagnostic Performance of AB-MRI According to Clinical Indication and Study Population.

Ref.	First Author	Study Design	Population	AB-MRI Protocol	Acquisition Time (min)	Sensitivity (%)	Specificity (%)	Other Diagnostic Outcome
**Supplemental Screening/Dense or Intermediate-Risk Breasts**								
[9]	Kuhl	Prospective observational reader study	443 women; 606 AB-MRI cases	Pre-T1W + first post-T1W + subtraction (FAST) + MIP	3	100; 90.9 MIP only	94.3	CDR: 18.2/1000
[24]	van Grinsven	Multireader secondary analysis of first-round screening; extremely dense, mammography-negative breasts	518 AB-MRI; 83 cancers	High-temporal + high-spatial DCE T1W series acquired within 120 s after contrast	~3.5	84.3	73.9	Reading time 49.7 s
[14]	Edmonds	Retrospective sequential supplemental screening; dense breasts, otherwise average risk	2585 AB-MRI cases	Axial STIR + pre-contrast T1W FS + single early post-contrast T1W FS + subtraction + MIP	NR	NR	NR	AIR 17.4% vs. 7.8%; CDR 18.9 vs. 12.1/1000; PPV2 21.3% vs. 28.0%; PPV3 26.6% vs. 29.2%
[25]	Kizildag Yirgin	Prospective feasibility screening study; dense breasts	238 women	Axial T1W + axial T2W FS (TRIM) + first and second post-contrast dynamic phases + subtraction	7–10	NR	NR	CDR 0; PPV 0; recall 15.5%; biopsy rate 2.94%
[43]	Garwany	Comparative study; intermediate risk	60 women	Contrast-enhanced abbreviated protocol	~10	100	98	PPV 66.7%; NPV 100%
**High-risk/BRCA/familial-risk screening**								
[27]	Panigrahi	Prospective paired high-risk screening cohort	1052 AB-MRI cases	Pre-T1W FS + single postcontrast T1W FS	~3	81.8	97.2	BI-RADS assessment changed by additional FDP-MRI sequences in 3.4%; CDR 13.3/1000
[17]	Naranjo	Retrospective multireader study; BRCA1/2 carriers	292 women; 427 AB-MRI cases	FAST alone vs. FAST + T2W	~4/~8	FAST: 92; FAST + T2W: 90	FAST: 71; FAST + T2W: 83	NPV 99% for both abbreviated protocols
[44]	Šupe Parun	Screening cohort with familial breast cancer history	178 women	TIRM + pre-contrast 3D T1W FS + two post-contrast 3D T1W FS	NR	91.7	94.6	PPV 55.0%; NPV 99.4%
[28]	Zaza	Retrospective high-risk screening audit	413 women	Pre-contrast T1W FS + single early post-contrast T1W FS + T2W FS + subtraction + MIP	~8	100	85.7	CDR 19.4/1000; PPV3 14.3%; NPV 100%
**PHBC surveillance**								
[29]	Fonseca	Randomized surveillance trial; women with PHBC	104 AB-MRI cases	Pre-T1W + first post-T1W + subtraction/MIP	~3	100	~78.8	CDR 48.1/1000; recall ~25%; biopsy rate 18.3%; PPV3 26.3%
[30]	Kim	Retrospective propensity-score matched PHBC surveillance	726 AB-MRI cases	T2W + pre-T1W + first post-T1W + subtraction/MIP	~8–9	100	93	CDR 21/1000; PPV3 61%; interval cancer rate 0/1000
[16]	Mun	Retrospective PHBC cohort; propensity-matched recurrence/nonrecurrence analysis	1002 screened; 21 recurrences + 42 matched controls	FAST → + ultrafast MIP → + delayed T1W → +T2W/DWI	NR	76.2–90.5; FAST 81	88.1–92.9; FAST 88.1–90.5	Accuracy 85.7–90.5%; FAST accuracy 85.7–87.3%
**Preoperative/pre-treatment staging**								
[32]	Hajri	Preoperative cancer cohort	228 women	MIP alone vs. FAST + MIP	~5	MIP 87.5; FAST + MIP 97.7	NR	NR
[45]	Arian	Cross-sectional, biopsy-proven cancer before initiation of NAC	54 women	Pre-T1W + two postcontrast T1W FS + subtraction + MIP	~4	NR	NR	Detection 100%; size assessment 90.7%
[34]	Zarb	Retrospective ILC-enriched diagnostic cohort	35 AB-MRI cases; 20 ILC + 15 negative controls	Pre-contrast axial T1W THRIVE FS + first and second post-contrast axial T1W THRIVE FS + subtraction + MIP	4.72	100	Reader I: 73.3; Reader II: 53.3	AUC 0.920/0.922; lesion localization 60–80%
	**Neoadjuvant therapy response**							
[18]	Dornelas	Single-center cross-sectional post-NAC cohort	210 women	I: Pre-contrast + first post-contrast T1W + subtraction + MIP; II: I + second post-contrast phase; III: II + third post-contrast phase	3.3/4.8/9.7	I: 83.2; II: 91.6; III: 91.6	I: 84.6; II and III: 81.3	Acquisition time: 3.3/4.8/9.7 min
[15]	Lo Gullo	Retrospective single-center post-NAC cohort	478 women	T2W + pre-T1W + early post-T1W	NR	NR	82.1/85.4 depending on pCR	No significant difference in PPV, NPV or accuracy; interpretation time 39–44 s
[35]	Chen I	Retrospective post-NAC multireader study	237 women; 92 pCR	Pre-contrast T1W FS + first post-contrast T1W FS + subtraction + MIP	NR	76.4	63.1	pCR prediction AUC 0.697; κ 0.64 vs. pathology
[19]	Chen Y	Multicenter retrospective early-response study	359 women	T2W + early DCE phase (~20 s) + DWI (Protocol 4)	6.2–7.6	73.3–80.6	78.5–88.1	AUC 0.876–0.880; accuracy 79.2–84.0
[38]	Spriet	Single-center mixed retrospective/prospective TNBC cohort; NAC + pembrolizumab	36 women	Ultrafast DCE-MRI, not conventional AB-MRI	NR	83	69	Agreement with FDP-MRI 0.96; sensitivity/specificity 78/69%
**Postoperative diagnostic**								
[41]	Karam	Prospective postoperative diagnostic study	47 women; 62 lesions	T1W/T2W + pre/early postcontrast + subtraction ± DTI	~6/~10	NR	NR	DTI sensitivity: MD 96.4%; FA 78.6%; AD 82.1%; RD 85.7%
[46]	Lee S	Retrospective multireader; selected lesions after previous breast cancer surgery	105 women/122 lesions	Classic: pre + two post-T1W; modified: + delayed T1W + T2W	2–3/8–10	Classic 76.8; modified 87.5	Classic 55.3; modified 52.7	AUC 0.70 vs. 0.76 (modified)

Abbreviations: AB-MRI, abbreviated breast magnetic resonance imaging; AD, axial diffusivity; AIR, abnormal interpretation rate; AUC, area under the receiver operating characteristic curve; BI-RADS, Breast Imaging Reporting and Data System; BRCA, breast cancer susceptibility gene; CDR, cancer detection rate; DCE, dynamic contrast-enhanced; DTI, diffusion tensor imaging; DWI, diffusion-weighted imaging; FA, fractional anisotropy; FAST, first post-contrast acquisition subtracted; FDP-MRI, full diagnostic protocol magnetic resonance imaging; FS, fat-saturated; ILC, invasive lobular carcinoma; MD, mean diffusivity; MIP, maximum intensity projection; NAC, neoadjuvant chemotherapy; NPV, negative predictive value; NR, not reported; pCR, pathologic complete response; PHBC, personal history of breast cancer; PPV, positive predictive value; RD, radial diffusivity; STIR, short tau inversion recovery; T1W, T1-weighted; T2W, T2-weighted; THRIVE, T1-weighted High-Resolution Isotropic Volume Examination; TIRM, turbo inversion recovery magnitude; TNBC, triple-negative breast cancer.

**Table 3:** Synthesized indication-specific AB-MRI framework and clinical trade-offs.

Clinical Indication	Target Population	Primary Diagnostic Objective	Potential AB-MRI Protocol Composition	Additional Components	Clinical Trade-Offs & Limitations	Evidence Maturity
Supplemental Screening	Dense breasts, intermediate risk	Early cancer detection	Pre-contrast T1W + 1st post-contrast T1W + Subtraction + MIP (FAST)	T2-weighted imaging/DWI	Higher recall and biopsy rates; limited lesion characterization	Relatively strong
High-Risk BRCA Screening	BRCA1/2 carriers, high familial risk	Early cancer detection	Pre-contrast T1W + 1st post-contrast T1W + T2W + Subtraction/MIP	Ultrafast DCE or DWI	BPE can cause false positives; T2W helps lower unnecessary recalls.	Moderate
PHBC Surveillance	Personal history of breast cancer (PHBC)	Detection of ipsilateral recurrence and second primary cancer	FAST-based protocol: pre-contrast + early post-contrast T1W + subtraction/MIP	T2W, DWI, ultrafast or delayed phase when clinically indicated	Additional sequences may provide only modest incremental benefit in routine surveillance	Moderate
Postoperative Diagnostic	Patients with a suspicious postoperative clinical or imaging finding	Differentiation of recurrence from benign post-treatment changes	Early DCE + subtraction/MIP ± T2W	DWI/DTI	Post-treatment changes may mimic malignancy; specificity is critical	Limited
Preoperative Staging	Newly diagnosed breast cancer	Mapping disease extent and multifocality	Pre-contrast T1W + early post-contrast T1W ± additional post-contrast phase(s) + subtraction/MIP	T2W and/or DWI	Risk of underestimation and false positives; management—changing findings require confirmation.	Limited
NAC Response Evaluation	Patients undergoing or completing neoadjuvant systemic therapy	Early response prediction and/or residual disease assessment	Pre-contrast T1W + early post-contrast DCE ± additional phase(s)	T2W and/or DWI	Performance varies by subtype and response pattern; DWI may improve accuracy	Limited/emerging

## Data Availability

No new data were created or analyzed in this study. Data sharing is not applicable to this article.

## Abbreviations

The following abbreviations are used in this manuscript:

AB-MRI	Abbreviated breast magnetic resonance imaging
ACR	American College of Radiology
AD	Axial diffusivity
AI	Artificial intelligence
AIR	Abnormal interpretation rate
AUC	Area under the receiver operating characteristic curve
BI-RADS	Breast Imaging Reporting and Data System
BPE	Background parenchymal enhancement
BRCA	Breast cancer susceptibility gene
CDR	Cancer detection rate
DBT	Digital breast tomosynthesis
DCE	Dynamic contrast-enhanced
DCIS	Ductal carcinoma in situ
DTI	Diffusion tensor imaging
DWI	Diffusion-weighted imaging
FA	Fractional anisotropy
FAST	First post-contrast acquisition subtracted
FDP-MRI	Full diagnostic protocol magnetic resonance imaging
FS	Fat saturation
HER2	Human epidermal growth factor receptor 2
HR	Hormone receptor
ILC	Invasive lobular carcinoma
MD	Mean diffusivity
MIP	Maximum intensity projection
MRI	Magnetic resonance imaging
NAC	Neoadjuvant chemotherapy
NME	Non-mass enhancement
NPV	Negative predictive value
NR	Not reported
pCR	Pathologic complete response
PHBC	Personal history of breast cancer
PPV	Positive predictive value
RD	Radial diffusivity
STIR	Short tau inversion recovery
T1W	T1-weighted
T2W	T2-weighted
THRIVE	T1-weighted high-resolution isotropic volume examination
TIRM	Turbo inversion recovery magnitude
TNBC	Triple-negative breast cancer
